# The Dietary Use of Pigeon Pea for Human and Animal Diets

**DOI:** 10.1155/2022/4873008

**Published:** 2022-01-24

**Authors:** Belete Kuraz Abebe

**Affiliations:** Department of Animal Science, Werabe University, P.O. Box 46, Werabe, Ethiopia

## Abstract

Pigeon pea (PP) [*Cajanus cajan* (L.) Huth] plays an important role in preserving poor smallholders' major source of income in the tropics and subtropics by improving food and feed security, particularly protein intake. In the meantime, protein deficiency is frequent in tropical and subtropical regions due to rapidly increasing human populations and the high cost of animal-origin proteins. As a result, pulse crops should be their primary source of protein. Among these, PP is the most important pulse crop utilized as a food component in rain-fed agricultural conditions with the lowest costs, and it is the best source of protein supplements in typical cereal-based diets to fill the nutritional deficit. Despite this, it is the world's least-used pulse crop. Therefore, the primary goal of this review was to provide and synthesize scientifically confirmed and up-to-date information on the dietary usage of pigeon pea for food and feed. Protein, carbohydrates, minerals, vitamins, and essential amino acids are all present in reasonable amounts in both mature and immature PP seeds. PP has the most potential for usage as food and feed, and its nutrients are comparable to those of soybeans and maize. PP's green leaves, roots, seeds, and pods are high in phenolic compounds, which have anti-inflammation, antibacterial, antioxidant, anticarcinogenic, and antidiabetic properties, as well as the ability to cure diseases like measles, smallpox, chicken pox, sickle cell anemia, fever, dysentery, hepatitis, and antimalarial medications for the body. Furthermore, the addition of pigeon pea and its by-products improves ruminant and nonruminant animal feeding performance significantly. In general, PP products such as dried grain, fresh (aerial portion), and green pods are used as a low-cost (low-cost) source of high-quality and quantity of protein food and feed for tropical and subtropical populations' livelihoods.

## 1. Introduction

In tropical and subtropical climates, pigeon pea (PP) [*Cajanus cajan* (L.) Huth] is the most prevalent fast-growing and adaptable pulse crop [1]. Cajan pea, no-eyed pea, and tropical green pea are all names for PP, which belongs to the Fabaceae family [2]. The total land area for PP production is expected to be 46,000 km^2^, with India accounting for 92% of that [3]. It is a drought-resistant pulse crop that thrives in locations with temperatures ranging from 20 to 40 degrees Celsius and less than 625 millimeters of annual rainfall, where other crops such as maize fail [4, 5]. It has a deep tap-root structure that allows it to collect water and other nutrients from the secondary soil profile, allowing plants to thrive during dry seasons. Due to its high drought resilience, PP can be regarded as the most significant pulse crop for food and feed security in dry places, where rainfall is scarce, making it a good crop for smallholder farmers in these locations [6]. PP provides green forage for animal feed when other forage crops have vanished due to a lack of rainfall during the dry season [7]. PP is a tropical perennial pulse crop that includes a good amount of carbohydrates, proteins, vitamins, minerals, and vital amino acids, and its seeds can be eaten fresh (immature) or dried (mature) [8]. PP is well known for delivering food proteins in rain-fed agriculture, which is typically farmed in low-cost places in impoverished nations [9, 10], and it considerably improves food and feed security in Africa, Asia, and South America [11].

PP seeds contain about 20–22 percent protein and appreciable amounts of essential amino acids and minerals [12]. It is the world's fifth-most well-known pulse crop and India's second [13]. It also contains a lot of crude fiber, minerals (both macro and micro), and vitamins [14]. Africans consume over 65 percent of the world's farmed PP plants [15]. PP is a significant pulse crop for food and feed, including large amounts of carbohydrates, protein, essential amino acids, fiber, vitamins, and minerals [12, 16]. It is the most important animal feed ingredient in West Africa. PP is sensitive to frost, soil salinity, and water logging, although it can grow in a variety of soil types with pH ranging from 5 to 7 [4, 17]. PP is beneficial to food and animal feed in a variety of ways. Dhal (split seed), mature and immature seeds, for example, are vital for cereal-based diet nutrition in humans. The developed seeds of PP and its by-products are vital for animal feed [18]. Furthermore, the leaves, pods, and seed by-products of PP contain a sufficient amount of protein for animal feed [19]. Similarly, in Hawaii, a chicken feed blend of PP seed and maize grain seed was successful [20]. Honey bees are actively fed PP, according to Orwa et al. [21], and they create a characteristic greenish-colored honey in the comb. Smallholder farmers in the tropics and subtropics employ PP products like dry grain, green pods, and pod husks as an inexpensive protein source [22].

In addition to its food and feed value, PP's green leaves, roots, seeds, and pods contain a large number of phenolic compounds, which offer a number of health benefits [23, 24]. Furthermore, the dietary elements of PP are regarded as important for human nutrition, and it is clear that PP consumption is linked to a reduction in a variety of human disorders [25, 26]. PP is becoming a critical remedy for protein deficiency in underdeveloped countries, as animal-based proteins have grown prohibitively expensive. In the meantime, demand for proteins, vitamins, and a vital mineral has increased. Despite the fact that PP has the potential to improve the livelihoods of tropical and subtropical smallholder farmers by increasing food accessibility and protein absorption, it remains the world's least-used pulse crop [27]. In this regard, multiple researches have been undertaken in various parts of the world in order to identify the nutritional uses of PP. However, the use of PP and its by-products in human and animal diets has not been thoroughly investigated or collated. Therefore, the goal of this review was to provide and synthesize scientifically confirmed and up-to-date information on the dietary usage of pigeon pea for food and feed.

## 2. Review Method

This narrative review is based on theories and empirical findings, with saturated information extracted. Experiments and scientific findings were utilized to filter data and focus on recent publications. The period of search for this script is from June 01, 2021, to October 30, 2021. The script's literature search focused primarily on the dietary use of pigeon pea for food and feed. The information provided by Google Scholar, as well as peer-reviewed major indexers such as Web of Science, PubMed, and Scopus, was used to extract useful information during this review script. Thus, key words such as “pigeon pea cultivation,” “nutritive profile of pigeon pea,” “role of pigeon pea as food,” “role of pigeon pea as feed,” and “therapeutic uses of pigeon pea” were used to find journal articles, papers, books, and symposia. The next section of this script will go through some concepts and experimental settings from the perspectives of PP production, pigeon pea nutritional profile, and dietary usage of pigeon pea for food and feed, and pigeon pea medicinal uses.

## 3. Pigeon Pea Production or Cultivation

PP was first grown in India, but it is the world's least utilized pulse crop. It does, however, provide certain benefits for smallholders in the tropics and subtropics [27]. India, Myanmar, Malawi, Kenya, and Tanzania are the world's top PP producers. Asia, Africa, and America accounted for 79.1%, 17.6%, and 2.5 percent of the world's total production potential, respectively [28]. PP can be grown in a variety of agroecological zones and is suited to tropical climates. According to Kimani [29], the potential output of PP is 5 ton/ha. Nonetheless, in smallholder farmers' fields, the likely yield of PP is less than one ton/ha [10]. The paucity of sophisticated gene variations, reduced usage of gene banks, inadequate husbandry, and exposure to salinity and water logging pressures are the key causes for the relative yields of PP seeds. In arid areas of tropical countries, PP is a critical pulse crop for food and feed security, and it is the only pulse crop that produces some grains when other pulse crops and cereals get wilted and dried due to moisture stress [30].

**Figure 1:**
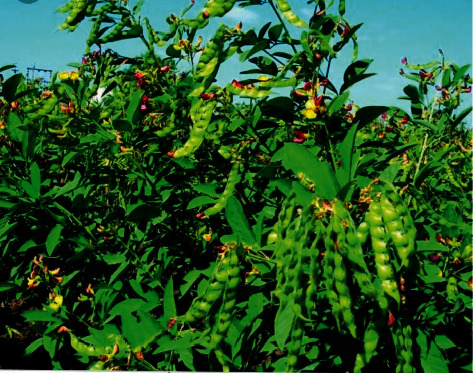
The blossoms of the pigeon pea pod [31].

The early-blowing variants of PP, as shown in Figure 1, necessitate a density of 300,000 plants per hectare. Late-rising varieties, on the other hand, necessitate 40,000–50,000 plants per hectare for optimal yields. For village and bund cultivation (producing) systems, PP plants are maintained for more than a year and reach a height of more than 3 meters.

**Figure 2:**
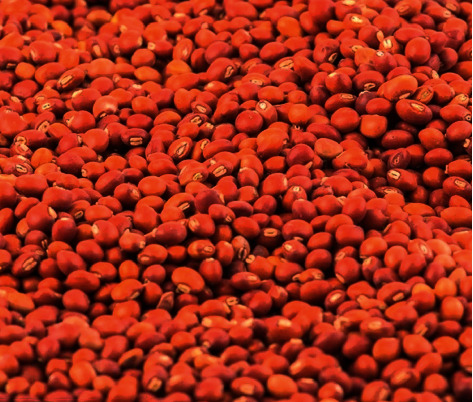
Harvested matured (Dry) pigeon pea seeds [14].

For a village production system, three to four PP seeds might be placed on a single hill, and the plants would generate a large number of twigs on both sides of the bund. In other words, a field with a track record of strong soil fertility and drainage should be selected for periurban commercial cultivation. For soil shortages, a 100 kg/ha diammonium phosphate application and other soil adjustments are recommended prior to sowing. Plant PP seed, as shown in Figure 2, at the beginning of the rainy season, row to row (100 cm) and plant to plant (50 cm) spacing, at a depth of 5 cm, and tightly covered by soil [14].

## 4. The Nutritive Profile of Pigeon Pea

PP is a good source of carbs, proteins, fats, and minerals, and it makes a significant contribution to human and animal nutrition. Carbohydrates, proteins, minerals, vital amino acids, and vitamins are abundant in both mature (dry) and immature seeds of PP (Table 1). PP seeds have a protein level that ranges from 18 to 25% [33]. Fiber, minerals, and vitamins (i.e., Vitamin C and E, thiamine, riboflavin, and niacin) are all present in appropriate amounts in the seeds of PP [14]. The primary components of PP seeds include carbohydrates, lipids, and proteins. PP is a common diet for many individuals in underdeveloped nations due to its high digestible percentage of protein and carbohydrates. It makes a significant contribution to meeting the nutritional needs of smallholders in terms of fiber, ash, fat, and minerals [19]. According to Miller et al. [34], PP seeds contain a large quantity of vitamin B and carotenes, both of which are commonly low in cereal crops; as a result, they can be used as a supplement to standard cereal-based diets. Furthermore, even though PP seeds are deficient in sulfur-containing amino acids, they contain a significant amount of lysine. Faris and Singh [19] showed that the seed of PP could improve the gain of lysine (in rice and wheat-based diets) and threonine, leucine, and isoleucine in wheat-based nutrition.

### 4.1. Carbohydrates

The main carbohydrates contained in PP are soluble sugars, starch, and dietary fiber. According to a study by [35], the components of total soluble sugars identified in PP carbohydrates range from 33.23 mg/g to 60.80 mg/g, whereas the levels of soluble sugar in wild PP species range from 38.10 to 40.54 mg/g. Sharma et al. [36], on the other hand, found a 31 mg/g soluble sugar concentration in dried PP seeds. In other words, the levels of starch detected in mature and immature seeds range from 272.70 mg/g to 521.28 mg/g, and in wild cultivars, they range from 90.46 mg/g to 191.27 mg/g [12]. Furthermore, the starch content of PP seeds varies between 41 and 53 percent, according to Trinidad et al. [37] and Sharma et al. [36]. PP also has a high amount of starch and fibers that are resistant to digestion in the small and large intestines, allowing bacteria to ferment and produce short-chain fatty acids [38]. Dietary fiber in PP, both soluble and insoluble, has been shown to lower blood cholesterol and glycemic response. PP roots contain 21.8 and 19.4 g/100 g of soluble and insoluble fibers, respectively [39], and PP roots play a major role in cholesterol-reducing effects in particular [40].

### 4.2. Proteins

Proteins are the second most important component of PP, with 21.7 g/100 g of protein in ripe (dry) seeds, as shown in Table 1 [12]. In other words, Sekhon et al. [35] reported that the total component of soluble protein detected in PP ranges from 170.37 to 251.16 mg/g. Furthermore, many experts have reported varying protein constituents in various PP cultivars. For example, according to numerous studies, the total protein component in different PP cultivars ranges from 18.8 percent to 25.97 percent [36, 41, 42]. Protein values in hard to roast seeds ranged from 19.06 to 28.89 percent, while protein values in easily roasted seeds ranged from 21.01 to 29.24 percent [43]. According to some data, the protein from PP leaves has both preventive and curative properties in the fight against chloroform-induced hepatotoxicity, which is attributed to their antioxidative defense mechanism [44].

### 4.3. Fatty Acids

In mature and immature PP seeds (Table 2), palmitic acid is the predominant saturated fatty acid, accounting for 15 to 25 percent, 20 to 40 percent, and 26 to 30 percent of neutral lipids, glycol-lipids, and phospholipids, respectively. Furthermore, Ade-Omowaye et al. [46] observed that linoleic acid was the most abundant polyunsaturated fatty acid in PP, while caprylic, lauric, oleic, and eicosanoid acids were only found in trace amounts.

### 4.4. Minerals and Amino Acids

According to Table 1, PP seeds (mature and immature) and by-products contain an adequate amount of macro- and microminerals, as well as important amino acids. According to Swaminathan [47], chronic magnesium deficiency can lead to diabetes, high blood pressure, and heart attacks. On the other hand, calcium is an essential macromineral for bone and tooth health as it promotes bone development and growth. The calcium (Ca) and magnesium (Mg) content of PP seeds is similar to that of other legumes, such as soybeans (215 mg/100 g). As a result, the health effects of mature and immature PP seeds for various Ca and Mg deficiency illnesses differ [48]. When used in cereal-based diets, the mature and immature seeds of PP include a reasonable amount of essential amino acids such as tryptophan, threonine, isoleucine, leucine, lysine, histidine, valine, and methionine plus cystine [46]. As a result, they have a great economical supply of amino acids, which, when combined with other food crops, could improve diet quality by lowering the risk of protein deficiency in underdeveloped nations.

### 4.5. Antinutritional Factors

Dietary inhibitors such as phytolectins, polyphenols, and enzyme inhibitors are found in small levels in the mature seed of PP (Table 3). According to Onwuka [50], the ripe seed of PP contains a lot of food inhibitors such as trypsin, chymotrypsin, alkaloids, and tannins. In different PP cultivars or hues, the components of dietary inhibitors contained in PP seeds are varied. Red-seeded PP genotypes, for example, have three times the amount of polyphenols as white-seeded genotypes. Antinutritional elements such as amylase inhibitors, protein inhibitors, and phytic acid are abundant in them [51]. Tannin-protein complexes seen in PP cultivars, on the other hand, are linked to and expelled with feces and are responsible for decreased crude protein degradability, reduced amino acid accessibility, and increased fecal nitrogen [52]. Although the amount of polyphenolic compounds found in PP is small (in comparison to soybean and beans), the presence of these compounds in PP genotypes causes digestive enzymes like trypsin, chymotrypsin, and amylase to be inhibited, which can cause problems when large amounts of PP products are consumed [53]. The best strategies for reducing these chemicals include chemical soaking, germination, and boiling of PP [54, 55].

## 5. Pigeon Pea as Food

PP is consumed in a variety of ways as a food (Table 4). Meanwhile, because animal-origin proteins have grown too expensive for low-income tropical and subtropical smallholders, protein deficiency is becoming more widespread. As a result, pulse crops should be their primary source of protein. PP is the most important pulse crop used as a food component in rain-fed agricultural conditions with the lowest costs, and it is the most important source of protein supplements in traditional cereal-based diets to fill the nutritional gap of protein supplements in the tropics and subtropics [14, 59].

## 6. Therapeutic Uses of Pigeon Pea

PP's leaves, seeds, and roots are used for a variety of therapeutic purposes (Table 5). For example, green leaves and pods of PP are used for therapeutic purposes in various nations. According to multiple studies, the tiny extracts from the leaves of PP contain a high level of antioxidants and are widely used in the treatment of diseases such as diabetes, fever, dysentery, hepatitis, and measles [37, 75, 76]. Similarly, PP root extracts are utilized as a fever reliever and an anthelminthic; fresh seeds are vital for male urinary system problems; and juvenile (immature) PP seeds are recommended for kidney problems [27]. They are necessary for the healing of wounds and sores by stopping the bleeding, as well as the treatment of various lung and chest disorders.

Pigeon pea seed extract has also been found in clinical investigations to aid in the reduction of red blood cell sickling, suggesting that it may be beneficial for people with sickle cell anemia. Sickle cell anemia can be treated with pigeon peas. Many sickle cell disease patients in Chhattisgarh utilize pigeon pea to decrease erythrocyte sickling [77]. The PP plant was discovered as a traditional medicinal herb used to treat anemia in northern and south-eastern Côte d'Ivoire [78]. For example, PP seed extracts have been demonstrated to reduce red blood cell sickling, making them potentially beneficial for people with sickle cell anemia. Its extract appears to be safe, simple to administer, and helpful in reducing painful crises, and it may be especially useful for sickle cell anemia sufferers [79].

According to multiple studies, the leaves, seeds, and roots of PP have a significant degree of anti-inflammation and antibacterial characteristics [48, 80], as well as a variety of health advantages due to their low saturated fat content and high levels of key minerals and micronutrients [81]. Antioxidant, anticarcinogenic, anti-inflammatory, and antidiabetic activities are also present [82]. According to Steven and Ehrlich [83], a few highly industrialized countries are interested in using organic medicines for a portion of their health care. In general, the leaves, roots, and seeds of PP have shown to be beneficial in the treatment of ulcers, inflammations, measles, hypertension, smallpox, chicken pox, sickle cell anemia, and other conditions in various parts of the world [77, 78, 80, 84–87]. Aside from the aforementioned uses, the seeds of PP are also used to treat diabetes, diarrhea, hepatitis, and antimalarial medications [75, 88].

## 7. Pigeon Pea as Feed

Several studies have found that PP and its by-products are excellent fodder (forage) species that are necessary for animal feed (Table 6). The PP plant's dry leaves, pods, pod husks, and by-products are critical for livestock rationing, because they provide a large amount of yield (biomass) with a high amount of nitrogen under low effort conditions, as well as palatable forage (grazing) vegetation for smallholder farmers in the tropics and subtropics during periods of water scarcity [20, 27, 90]. As a result, PP is the most significant pulse crop for animal feed additives, particularly in areas where other pulse crops (such as soybeans) struggle to grow [90]. Fresh PP pasture has a crude protein content ranging from 15 to 24 percent [91, 92]. In a number of species, the high biomass output (productivity) and nutritious content of PP fodder resulted in good live weight growth. For example, in a study conducted in Hawaii, pure PP forage provided superior weight gain (280 kg/ha/year) to mixed grass pasture (181 kg/ha/year) for more than 6.5 months of grazing periods [93], and Rao et al. [94] reported that young cattle (calves) grazing the PP crop intensively in the late summer had an average body weight gain/d closer to 1.0 kg/d.

On the other hand, sheep selected fresh PP plants (forage) from eight browse forge species [95], and the amount of PP meal supplied to layers enhanced (improved) their laying performance (up to 7.5 percent), as well as their egg yolk color score [96]. Goats outperformed sheep when it came to using PP leaves as a feed additive [97]. Dry PP leaves are an excellent substitute for other forage species (alfalfa) in chicken feeds as a source of carotenes and other key elements. Furthermore, according to Saxena et al. [12], PP's fresh and dried leaves are important fodder supplements, and its crop leftovers after threshing are used as dairy cattle feed. Seed coatings and powders from Dhal mills are important by-products of PP seeds for cows, poultry, and pig diets. Sugui et al. [98] investigated whether PP could be a cheap and efficient way to feed chickens.

In chicken and pig feeds, the whole seed, pods, pod husks, hay, and milling by-products of PP could be a superior replacement for soybeans and maize [99]. Similarly, the leaves of PP are occasionally used to replace alfalfa in the nutrition of large and small ruminants, and the seeds and processing by-products are also essential for animal feed [20, 100]. As previously stated, PP and its by-products have a wide range of uses in animal nutrition. Fresh leaves, pods, hay, and pod husks, for example, are edible protein-rich fodders for animals. PP seeds are also useful for providing carotene and other nutrients to poultry and honey bee diets [21, 101]. The dry matter (DM) digestibility of PP hay varies between 50 and 60% [102], and the in vivo dry matter digestibility of PP hay is similar to that of cowpea hay (55–56 percent) [103]. Foster et al. [103] found that the hay of PP had fewer digestible elements than those of other pulses like groundnut and cowpea. Similarly, Foster et al. [104] and Veloso et al. [105] found that when compared to other hay of low quality fodders, the fresh hay and leaves of PP are distinguished by low ruminal dry matter, neutral detergent fiber, and nitrogen disappearances. The dry matter (DM) content of PP pod husks is roughly comparable to the seed content, according to Ferreira et al. [106]. The pod husks, on the other hand, are high in crude fibers and low in crude proteins.

According to Whiteman and Norton [92], adding tiny amounts of other high-quality forages to cattle feed can boost the nutritional content of PP pods and pod husks. Broilers could achieve a high inclusion rate of PP seeds as a replacement for sesame cake (up to 12 percent) [107]. However, if their inclusion rate in their meals exceeds 20%, their performance may suffer [108, 109]. With the exception of fermentation, most processing methods improve broiler development performance [110]. In general, raw PP seeds should make up 10% of broiler meals. Raw PP seeds at a 30% level in layer feeds have been shown to reduce hen egg production per day [111, 112].

According to Singh and Kush [113], PP plants have the potential to produce a large amount of yield (biomass) ranging from 40 to 57.6 ton/ha. Similarly, Whiteman and Norton [92] found that around half of the PP yield is edible and useful for livestock feed. The adoption of the chopping procedure in PP fodder boosts sheep feed intake by 60% [95]. Several studies found that the voluntary DMI of sheep on PP leaf-based rations was 2.5 percent of animal body weight [9] and 3.5 percent of body weight on PP hay-based rations in similar species [102]. Animals may eat the leaves and young pods of PP, and it supplies high-quality fodder. For animal feed production, PP grows better when intercropped with sorghum and millets [1, 114]. Rao and Northup [94] discovered that yearling cattle gained an average of 1.0 kg/d when fed vigorously grazed PP forages. PP fresh forage biomass yields typically range between 20 and 40 ton DM/ha, and PP is classified as a high biomass (yield) producer pulse crop [7]. PP seeds and by-products are generally significant for livestock feed, and the seeds provide a high-quality supply of proteins, with up to a 20% dry matter (DM) basis for lactating cow diets. Furthermore, uncooked (raw) seeds can be included at a rate of less than 30% in goat rations and 20% in growing pig rations. In broiler and layer rations, about 10% raw PP seeds and 20% processed seeds can be added [115].

## 8. Conclusions

The purpose of this narrative review was to discover the nutritional and phenolic components of pigeon pea, as well as their relevance in human and animal nutrition. In the meantime, protein deficiency is frequent in tropical and subtropical regions, owing to a rapidly growing human population and the high cost of animal-origin proteins. As a result, pulse crops should be their primary source of protein. PP is the most important pulse crop utilized as a food component under rain-fed agricultural circumstances, and it is the most important source of protein supplements in traditional cereal-based diets to fill the nutritional deficit. Protein, carbs, minerals, vitamins, and fundamental amino acids are all present at reasonable levels in both mature and immature PP seeds. PP has the most potential for usage as a food and feed source, and its nutrients are comparable to those of soybeans and maize. In addition to its dietary value, PP's green leaves, roots, seeds, and pods contain a large number of phenolic compounds, which have anti-inflammatory, antibacterial, antioxidant, anticarcinogenic, and antidiabetic properties, as well as the ability to treat diseases such as measles, smallpox, chicken pox, sickle cell anemia, fever, dysentery, hepatitis, and antimalarial medications. Furthermore, the addition of PP and its by-products improves the performance of ruminant and nonruminant animal nutrition significantly. In addition to the foregoing explanations, PP contains antinutritional substances that may have an adverse effect on nutrient bioavailability. Chemical soaking and cooking techniques, on the other hand, may help mitigate these antinutritional effects. Smallholder farmers in the tropics and subtropics use PP and its by-products, such as dry grain, green pods and pod husks, and hay, as a low-cost source of edible proteins for food and feed. As a result, significant emphasis should be put on the production and utilization of PP in traditional cereal-based diets in order to fight protein deficiency in developing countries.

## Figures and Tables

**Table 1:** Nutritional profile of immature and mature pigeon pea seed [32].

Nutrients	Immature seeds (g/100 g)	Mature seeds (g/100 g)
Carbohydrates	23.88	62.78
Fat	1.64	1.49
Protein	7.20	21.70
Vitamins (mg/100 g)		
Thiamine (B_1_)	0.40	0.64
Riboflavin (B_2_)	0.17	0.19
Niacin (B_3_)	2.20	2.96
Vitamin B_6_	0.68	0.28
Vitamin C	39.00	—
Vitamin E	0.39	—
Minerals (mg/100 g)		
Calcium	42.00	130.00
Iron	1.60	5.23
Magnesium	68.00	183.00
Manganese	0.57	1.79
Phosphorous	127.00	367.00
Potassium	552.00	1392.00
Sodium	5.00	17.00
Zinc	1.04	2.76
Amino acids (essential) (mg/g of protein)		
Tryptophan	—	9.76
Threonine	—	32.34
Isoleucine	—	36.17
Leucine	—	71.30
Lysine	—	70.09
Methionine + cystine	—	22.70
Valine	—	43.10
Histidine	—	35.66

**Table 2:** Mature (immature) seeds of PP fatty acid profiles [45].

Fatty acids (mg/100 g)	Quantity
Palmitic acid	236.00 ± 11.0
Stearic acid	40.95 ± 3.31
Oleic acid	78.55 ± 6.71
Capric acid	—
Lauric acid	—
Myristic acid	—

**Table 3:** Some dietary inhibitors in pigeon pea (leaf, stem, and seeds) [49].

Dietary inhibitors	Amount in g/100g
Alkaloids	2.65 ± 0.01
Phenolic	3.82 ± 0.15
Flavonoid	2.11 ± 0.03
Saponin	6.35 ± 0.96
Tannin	0.23 ± 0.01

**Table 4:** Edible parts of PP plants.

Edible portions	Way of consumption	References
Seed (whole grain)	It is possible to consume the mature (dry) seeds of PP by overnight soaking and cooking methods.	[56]
Germinated seed	After soaking the PP seeds in water, they germinate well and can be eaten raw or cooked.	[57]
Dhal	It is the dry seeds of the PP cotyledon without seed coat that are popular as they take a short time to cook and have good palatability when consumed by humans.	[19]
Green seeds as vegetable	Green seeds have a high amount of sugar and fat, and they have better protein and starch digestibility than mature dry seeds. They also have fewer protein inhibitors and higher amounts of iron and calcium than in mature dry seeds and Dhal.	[58]
Fresh pods	Fresh pods of PP are used as food after cooking, and they are used as salads.	[19]

**Table 5:** Medicinal uses of pigeon pea seeds, leafs, and roots.

Physiological activity	Functions (roles)	References
Hypocholesterolemic effect	PP seeds, leaves, and roots have significant amounts of saponins that are important for reducing high levels of cholesterol in the blood.	[60]
Antimicrobial effect	Extracts from the leaves of PP have a considerable amount of natural antimicrobial compounds like tannins, flavonoids, and alkaloids with important antifungal properties.	[61–63]
Hypoglycemic activity	PP is among the most efficient hypoglycemic curative plants, treating diabetes and its complications with various levels of hypoglycemic activity.	[64]
Hepatoprotective effect	The protein extracts from the PP plant are able to work against liver inflammation, reduce liver injuries and disease development, and its complications.	[65–68]
Cancer prevention	In humans, the anticancer chemical derived from the roots of PP can be used to treat breast and lung cancer cells.	[69, 70]
Anti-inflammatory effect	The presence of cajaninstilbene acid only in PP leaves along with its synthesized derivatives revealed strong slowing down activities on the release of inflammatory factors.	[71, 72]
Antihyperglycemic activity	Studies on the cooked PP seeds have shown a significant reduction in blood glucose levels.	[73]
Antidyslipidemic activity	Some statistical results on PP plants showed that they have significant amounts of antidyslipidemic activity in the body.	[74]

**Table 6:** Nutritional profile of pigeon pea and its by-products [89].

Parameter	Fresh (aerial portion)	Hay	Pods	Pod husks
Dry matter (% as fed)	24.4–49.7	88.8–91.8	87.3	93.0
Crude protein (% DM)	10.1–26.7	12.2–16.7	20.3	6.7
Crude fiber (% DM)	21.3–45.1	32.5	35.2	38.0
NDF (% DM)	37.2–62.9	78.6	-	-
ADF (% DM)	15.7–38.7	60.2	-	-
Lignin (% DM)	7.3–21.4	17.1	-	-
EE (% DM)	2.4–6.1	1.9	1.7	0.3
Ash (% DM)	4.0–8.8	3.9–5.3	3.3	4.1–5.8
GE (MJ/kg DM0	19.7–24.5	19.2	—	18.4
Macro minerals (g/kg DM)				
Calcium	4.6–10.8	—	—	9.7
Phosphorous	0.1–0.26	—	—	1.8
Potassium	9.1–20.8	—	—	—
Sodium	0.2–0.3	—	—	—
Magnesium	1.5–5.5	—	—	3.0
Micro (trace) minerals (mg/kg DM)				
Manganese	73–75	—	—	—
Zinc	23–54	—	—	33
Copper	7–12	—	—	13
Iron	181–244	—	—	744
Amino acids (% of protein)				
Arginine	5.7	—	—	—
Histidine	2.7	—	—	—
Iso-leucine	3.8	—	—	—
Leucine	6.6	—	—	—
Lysine	2.2	—	—	—
Phenyl-alanine	5.8	—	—	—
Valine	5.2	—	—	—

GE = gross energy; EE = ether extract; ADF = acid detergent fiber; NDF = neutral detergent fiber.

## Data Availability

The documents used to support the review of this article are available from the corresponding author upon reasonable request.
